# Tropane Alkaloids: Chemistry, Pharmacology, Biosynthesis and Production

**DOI:** 10.3390/molecules24040796

**Published:** 2019-02-22

**Authors:** Kathrin Laura Kohnen-Johannsen, Oliver Kayser

**Affiliations:** Technical Biochemistry, Department of Biochemical and Chemical Engineering, Technical University Dortmund, D-44227 Dortmund, Germany; Laura.Kohnen@tu-dortmund.de

**Keywords:** tropane alkaloids, scopolamine, cocaine, calystegine, chemistry, pharmacology, biosynthesis, biotechnological production

## Abstract

Tropane alkaloids (TA) are valuable secondary plant metabolites which are mostly found in high concentrations in the Solanaceae and Erythroxylaceae families. The TAs, which are characterized by their unique bicyclic tropane ring system, can be divided into three major groups: hyoscyamine and scopolamine, cocaine and calystegines. Although all TAs have the same basic structure, they differ immensely in their biological, chemical and pharmacological properties. Scopolamine, also known as hyoscine, has the largest legitimate market as a pharmacological agent due to its treatment of nausea, vomiting, motion sickness, as well as smooth muscle spasms while cocaine is the 2nd most frequently consumed illicit drug globally. This review provides a comprehensive overview of TAs, highlighting their structural diversity, use in pharmaceutical therapy from both historical and modern perspectives, natural biosynthesis *in planta* and emerging production possibilities using tissue culture and microbial biosynthesis of these compounds.

## 1. Introduction

**Figure 1:**
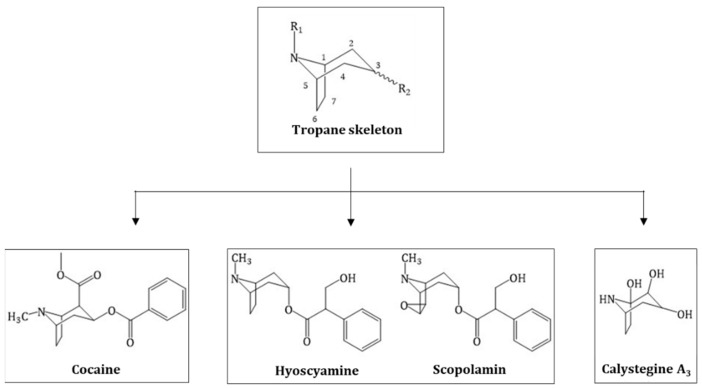
Structure of the tropane skeleton (green box) and the three major groups of TAs derived from this skeleton.

Alkaloids are naturally occurring compounds containing one or more nitrogen atoms. The name is derived from the basic nature of many members of this group, alkaloids from “alkaline-like”. The definition of alkaloids is complex as many nitrogen-containing molecules do not necessarily belong to this group. Biogenic amines or amino sugars, for example, are natural plant products and *N*-containing but not defined as alkaloids. Tropane alkaloids (TAs) are a specific class of alkaloid and can be more specifically defined as all molecules that possess a tropane ring system (Figure 1 Structure of the tropane skeleton (green box) and the three major groups of TAs derived from, green box) [1].

TAs are either esters of 3α-tropanole (tropine) or, to a lesser extent, 3β-tropanole (pseudotropine) and can be distinguished into three groups: TAs from Solanaceae plants like hyoscyamine and scopolamine, coca alkaloids like cocaine from *Erythoxylum coca* and the recently discovered calystegines group, which are polyhydroxylated nortropane alkaloids (NTAs) mainly occurring in Convolvulaceae, Solanaceae, Moraceae, Erythrocylaceae and Brassicaceae (Figure 1 Structure of the tropane skeleton (green box) and the three major groups of TAs derived from) [2]. In total, ~200 different TAs have been described [3].

Biosynthesis of the tropane ring system is homologous in organisms which produce these three TA classes. TA biosynthesis begins with the amino acids ornithine or arginine and their intermediate putrescine, continuing to the common *N*-methyl-Δ^1^-pyrrolinium cation precursor of all TAs. This is the branch point of cocaine, hyoscyamine/scopolamine and calystegine as well as nicotine biosynthesis [4].

Although all TAs have a high degree of structural similarity due to their tropane ring, the pharmacological effects of these compounds differ significantly. Cocaine and hyoscyamine/scopolamine are able to pass the blood-brain barrier and commit dose-dependent hallucination and psychoactive effects. Calystegines do not cause these effects due to their polarity as well as hydrophilicity and consequent inability to pass this barrier.

The cultivation of coca plants, the extraction of cocaine and production of other cocaine-containing drugs as well as their trade, with a few exceptions, is illegal and cocaine is the 2nd most frequently consumed illicit drug globally [5]. Due its legal designation, research has only been conducted on pathway elucidation in order to understand cocaine biosynthesis, however, research of large-scale commercial production has not been conducted (legally). As the calystegines are a newly discovered group of TAs without any pharmaceutical, medicinal or economic interest, little research has thus far been performed on this group of TAs. In contrast, the cultivation and production of scopolamine is of major economic interest due to its miscellaneous pharmaceutical applications. Indeed, global demand for this compound is increasing. Moreover, scopolamine is one of the Essential Medicines of the World Health Organization (WHO) [6]. Hyoscyamine and scopolamine are extracted from the *Duboisia* plants being cultivated on large plantations in Queensland, Australia [1]. Climate change and resulting new biotic and abiotic factors challenge the pharmaceutical industry to produce consistently high volumes of scopolamine. To overcome this issue, alternative production methods have been also tested.

This review seeks to provide a comprehensive overview of current knowledge on medicinal and pharmaceutical applications of TAs, a comparative analysis of TA biosynthesis and future strategies for elucidation of biosynthetic pathways, with special focus placed on the production of scopolamine as well as derivatives and enhancement of their production.

## 2. History and Chemical Elucidation of Tropane Alkaloids

The TAs cocaine, scopolamine and calystegines share a common tropane moiety. Nevertheless, these compounds cause very different physiological effects in humans. Cocaine manifests its effects in the synaptic cleft by inhibiting the dopamine, noradrenaline and serotonin reuptake while scopolamine acts as a competitive muscarinic receptor antagonist. The ingestion of both substances may lead to hallucinations and psychoactive effects or death [7,8]. Calystegines, on the other hand, are not absorbed into the central nervous system (CNS) due to their hydrophilicity and consequently, exhibit no psychoactive effects in humans [9].

### 2.1. Hyoscyamine and Scopolamine

TA producing Solanaceae plants are distributed globally. *Duboisia* plants are found in Australia and New Caledonia, while *Datura* plants, which had their origin in Asia and America, grow worldwide except in polar und subpolar climate zones. Members of the genus *Atropa* and *Hyoscyamus* have origins in Europe, Asia, as well as North Africa and were introduced to the USA, Canada and Australia [10]. All plants are simple to cultivate and readily found in nature, highly potent, and, consequently, have a long history in traditional medicines from different cultures. Until single compounds were isolated, whole plant or herbal preparations of these plants including extracts, ointments or teas were used for medicinal applications. Earliest reports of hyoscyamine or scopolamine-induced states of perception reach back into antiquity. Over 3000 years ago, *Mandragora* extracts were added to beer in Egypt to lower amounts of alcohol in these beverages. In Russia and China, *Datura* extracts and in Europe *Hyoscyamus* was added to enhance the thrilling effect of beer. A physician in Babylonia documented the analgesic property of the nightshades to reduce toothache [11]. In addition to hallucinogenic and analgesic effects, nightshades have a history of being used as poisons, for example, a wave of unexplainable mortality in the French high-society during was attributed to these plants [12]. In Australia, indigenous people exploited the TA-containing *Duboisia* plants for centuries for their cholinergic activity [13]. *Hopwoodii*, also called pituri, produces the alkaloid nicotine, which is arguably more widely popular for common use than the *Duboisia* plants *leichhardtii* and *myoporoides* [14].

Solanaceae plants have been given several names due to their historical and widespread use. These names often reflect the type of application and respective pharmacological action. *Datura stramonium*, which was introduced to European medicine by Romany immigrants, is called asthma herb due to its application in mitigating the symptoms of asthma. If *Datura* herb is smoked, a bronchorelaxation effect has been documented. Further names are “thornapple” after the hooked capsule or “horse poison” due to the toxic effects after ingestion on equines that are particularly sensitive to TAs [15]. The common *Datura* name jimsonweed is derived from Jamestown, a town in Virginia (USA) and it was reported that in 1676, settlers ingested this weed with fatal results. The intoxications were described and documented vividly leading to this additional name [10,16]. *Atropa* is named after the Greek goddess of fate and the goddess of the kingdom of the dead, Atropos. The species name belladonna is derived from its pharmacological effect, the mydriasis. Applying the extract into the eye, enflames beautiful eyes - with the disadvantage that the eye is unable to accommodate and one cannot see properly [11]. *Mandragora* and *Hyoscyamus* plants have historically played essential roles as the major active substances in the ointments of “witches” [17]. As TAs can easily be absorbed through the skin, it has been documented that the witches’ flying ointment was rubbed onto broomsticks, so the toxins were absorbed through the rectum and vagina. The intoxicating effects of these ointments have been reported as a feeling of lightness of being, followed by strong and vivid delirium and hallucinations. These effects were so pronounced that many users thought themselves to fly and the skill to fly was associated with witchcraft [18]. Solanaceae TAs were also used for love potion, exploiting the aphrodisiac qualities which could evoke sexual feelings [19].

The isolation and structural elucidation of TAs from Solanaceae plants began with the discovery of atropine. In 1832, this alkaloid was isolated by the German pharmacist H. F. G. Mein, however, he did not publish his results [20]. One year later, P. L. Geiger and O. Hesse (1833) published the isolation of atropine, a nitrogen containing, alkaline substance, from *Atropa belladonna* and *Hyoscyamus niger*. They described early investigations regarding the medicinal use, different isolation methods and chemical properties [21]. The stereochemical relation between atropine and hyoscyamine was elucidated by K. Kraut and W. Lossen almost fifty years later [22,23]. They were able to elucidate the reaction mechanism of the alkaline hydrolyzation of hyoscyamine and detected that the cleavage products of both, hyoscyamine and atropine, are tropic acid and tropine. From this it was concluded that atropine is the racemate of hyoscyamine [23]. A. Ladenburg (1879) discovered that the reverse reaction of the hydrolysis is possible by boiling the educts in hydrochloric acid and established a frequently used method of esterifying tropine with numerous organic acids [24].

### 2.2. Cocaine

The first reports of the use of cocaine date back to 3000 B.C. in Ecuador. The cultivation and chewing of coca plant leaves is assumed to have originated on the Eastern slope of the Ecuadorian or Peruvian Andes by the Inca peoples. Tribesmen traditionally chewed the leaves of the coca plant together with lime to release the alkaloids, both for spiritual purposes such as burial ceremonies or to give strength and energy and also to tolerate thin air high at high altitudes in the mountains. The coca plant and its invigorating effect was believed to be a mysterious gift of the gods. Before the Spanish soldiers entered South America, chewing coca leaves was reserved only for the tribal leaders. After the Spanish conquest of South America, its use was spread of over the continent and no longer socially limited. Cocaine was isolated for the first time in 1855 by F. Gaedecke. He published his results in the journal Archiv der Pharmacie and called the substance, isolated from coca leaves, Erythroxyline [25]. Working on cocaine was an interesting field but due to the limited access to plant samples available in Europe, little research could be conducted. Albert Niemann, who received enough supply of coca leaves for research, was able to proceed his study and improved the isolation process as well as the general knowledge of cocaine and its mode of action [26]. The chemical formula of cocaine was determined in 1862 by W. Lossen, who also dealt with the analysis of atropine. Subsequently, the first chemical synthesis and the elucidation of its chemical structure was achieved by R. Willstätter in 1898. About fifty years later, the stereochemistry was elucidated by the Swiss chemists E. Hardegger and H. Ott [27]. However, not all published reports on cocaine and its chemistry were scientifically or ethically correct. In 1885, S. Freud published his work “Über Coca” and recommended cocaine as an almost miracle medicine, with local anaesthetic properties, which is best for the treatment for postnatal depression and morphine addiction—a dangerous application for a substance with such high addictive potential [28].

### 2.3. Calystegines

Polyhydroxylated NTAs like calystegine do not show any psychoactive effects due to their inability to pass the blood-brain barrier based on their hydrophilicity. In addition, they exhibit minimal pharmacological activity. As a result, this class of NTAs have not found use in ancient medicines. Recently, researchers proposed that these compounds inhibit mammalian and plant glucosidases, although until now they do not have any pharmacological application and have received little research attention [29]. The first structures of polyhydroxylated NTAs were published in 1990 [2].

## 3. Pharmacology of TAs and Their Role as Drug Lead Substances

### 3.1. Scopolamine, Hyoscyamine and Anisodamine and Their Derived Drugs

**Figure 2:**
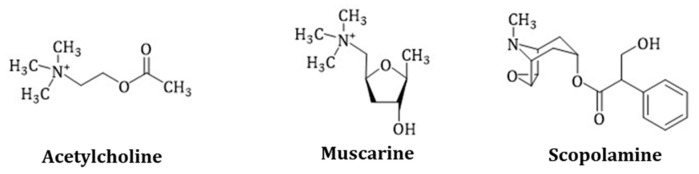
Comparison of the chemical structures of acetylcholine, muscarine and scopolamine. Scopolamine is protonated in the body due to the physiological pH and is present as a quaternary ammonium salt.

Hyoscyamine and scopolamine are widely used as anticholinergic drugs. They affect the central and peripheral nervous system as competitive, non-selective muscarinic acetylcholine receptor (mAChR) antagonists, that prevent binding of the physiological neurotransmitter acetylcholine. In humans, two acetylcholine receptor types are known: Muscarinic and nicotinic receptors, which are named after their agonists, muscarine (Figure 2) and nicotine. Muscarine is a poison of the toadstool mushroom *Amanita muscaria* and acts on the mAChR of the synapses like acetylcholine, with the difference that the acetylcholinesterase does not metabolize it.

The mAChRs are a subclass of the G-protein-coupled receptors (GPCRs) family, containing five subtypes (M1–M5). M1, M3 and M5 that are coupled with the stimulating Gq receptors and generate cytosolic calcium transients via the phospholipase C signalling pathway. M2 and M4, on the other hand, couple with the Gi protein and inhibit the adenylyl cyclase [7]. In particular, M1 receptors occur in the central nervous system and ganglia where they are involved in memory and learning processes. M2 receptors are found in the heart and are lower in abundance than M1 receptors. M3 receptors are involved in the contraction of the smooth muscles. M4 receptors were detected in the forebrain, hippocampus and striatum, they are likely involved in pain processes [30]. The physiological action of M5 receptors is not yet elucidated, however, it is assumed that these receptors are involved in brain microcirculation and mediate vasoconstriction, vasodilatation and activation of nitric oxide synthase [31].

TAs are absorbed from the gastrointestinal tract, rapidly distributed into the tissues and excreted predominantly through the renal system [32]. The short half-life in plasma and dose-dependent adverse effects limit the administration of scopolamine to transdermal application [33]. After absorption, scopolamine experiences a significant first-pass effect, because only a minor amount (2.6%) is excreted in the urine in the pharmacologically active form [34]. Cytochrome P450 enzymes seem to be especially involved in the metabolism of scopolamine by oxidative demethylation. Inhibition of CYP3A by ingestion of grapefruit juice prolonged the t_max_ and increased the AUC_0–24h_ value of scopolamine metabolization [33]. Additionally, it has been observed that scopolamine and its apo- and nor-metabolites are conjugated to glucuronide (glucuronidation) or sulphate during phase II metabolism for excretion into urine. Scopolamine and hyoscyamine do not accumulate in the human body, nor exhibit genotoxic or chronic toxicity, an adverse effect due to long-term exposure have not been reported (EFSA, 2013).

Occurring side effects of anticholinergic drug substances occur from inhibition of the parasympathetic nervous system. Symptoms include decelerated heart rate, dry mouth and reduced perspiration. At higher therapeutic oral doses, increased heart rate, inhibition of the respiratory tract secretory activity as well as bronchodilation and mydriasis have been observed. Sweating is also inhibited which is accompanied by a consequent rise in body temperature.

A dysfunction of muscarinic cholinergic system has been drawn in various diseases like depression, epilepsy, Parkinson’s and Alzheimer’s disease. Therefore, antagonists of the muscarinic system such as the TAs remain of great interest as potential lead CNS drug substances. In addition to the properties described above, hyoscyamine serves as an antidote against toxins such as organothiophosphates, for example the pesticide parathion (E605) [35]. The organic phosphorous derivates are used as insecticides and as nerve gases applied in military weapons. The antidote against scopolamine and hyoscyamine intoxications is physostigmine, a pyrolo-indole alkaloid. Physostigmine naturally occurs in the Calabar bean (*Physostigma venenosum*, Fabaceae) and acts as parasympathomimetic drug by reversible inhibition of the cholinesterase [36].

#### 3.1.1. Scopolamine

Scopolamine causes mydriatic, spasmolytic and local anaesthetics effects yet exhibits side effects which can be hallucinogenic and even lethal. The most important mode of application for scopolamine is transdermal, a technology which was developed as transdermal therapeutic systems (TTS) in 1981. Scopoderm TTS^®^ is the trade name for a scopolamine delivery system used in the treatment of motion sickness. During the Second World War, scopolamine was used to treat shell shock, psychoactive side effects and also motion sickness [13]. The drawbacks of scopolamine lay in the manifold peripheral and central nervous system side effects. To overcome these issues, scopolamine derivates have been developed, leading to its classification as a drug lead substance.

#### 3.1.2. Hyoscyamine and Atropine

Hyoscyamine and atropine have similar modes of action and effects as scopolamine. The pharmacological action of TAs is stereoselective, due to the difference of the stereoisomers concerning affinity and binding to muscarinic receptors. This results in different potency between *S*-(–)- and *R*-(+)- isomers of hyoscyamine: The *S*-(–)- isomer is estimated to be 30–300 fold more potent than the *R*-(+)- isomer [37]. The *S*-(−)-isomer of hyoscyamine is not stable and is racemized rapidly to atropine, which is a 1:1 mixture of the two forms. Atropine is very stable over time and hence, it used for medicinal applications instead of hyoscyamine. Both, atropine and scopolamine have a characteristic, dose dependent action on the cardiovascular system, which is clinically useful for resuscitation.

#### 3.1.3. Anisodamine

Anisodamine, which is isolated from *Anisodus tanguticus*, a Tibetan regional plant, is less toxic than atropine and scopolamine. It has a long tradition in folkloric Asian medicine especially in the treatment of septic shock by improvement of blood flow in microcirculation and also in various circulatory as well as gastric disorders with similar effects to atropine and scopolamine.

#### 3.1.4. Homatropine, Cyclopentolate and Tropicamide

Homatropine, the mandelic acid ester of tropine, is used in ophthalmology to evoke a more rapid and less paralytically effect than atropine. This is a major advantage over atropine and, consequently, homatropine was launched as a new mydriatic by Merck Darmstadt in 1883 as one of the first synthetic drugs [38]. Other modified mydriatic agents are cyclopentolate, which is used especially for paediatric eye examinations and tropicamide, which has been approved in ophthalmology since 2005.

#### 3.1.5. Trospium Chloride

Trospium chloride is a quaternary ammonium 3α-nortropane derivate esterified with benzylic acid. This synthetic anticholinergic is not able to cross blood-brain barrier and relaxes the smooth muscle in the bladder. Its main application is to treat urgency and reflex incontinence [39].

#### 3.1.6. Tropisetron

Tropisetron possesses a tropane skeleton but due to its mechanism of action it belongs to the serotonin receptor antagonist. It is applied to antiemetic therapy in cases of nausea and vomiting during chemotherapy and additionally as analgesic in fibromyalgia [40].

#### 3.1.7. *N*-butylscopolamine

To minimize adverse effects on the central nervous system, scopolamine has been modified by *N*-butylation and, in this form, it cannot longer pass the blood-brain barrier. *N*-butylscopolamine is used to treat abdominal pain from cramping, renal colic and bladder spasms [41]. Available dosage forms are as tablets or film-coated tablets (also available in combination with paracetamol), rectal suppositories (also available in combination with paracetamol) or solutions for injection and the according drug products are Buscopan^®^ or Buscofem^®^.

#### 3.1.8. Tiotropium Bromide, Ipratropium Bromide and Oxitropium Bromide

In traditional medicine, smoking of *Datura* leaves have been frequently used to treat asthmatic symptoms. Bronchodilation is caused by blocking of M3 receptors located on smooth muscle cells in the bronchi. Scopolamine and hyoscyamine are the TAs responsible for this effect. To reduce the adverse and intoxicating effects of this treatment, tiotropium bromide and ipratropium bromide were developed and are also administered by inhalation. Tiotropium bromide (Spiriva^®^; released on the market in 2005) is dominantly used in the treatment of chronic obstructive pulmonary disease (COPD) while ipratropium bromide (Atrovent^®^; released 1975) is used in the treatment of COPD (in combination with salbutamol, a β2-adrenergic receptor agonist) and asthma [42]. Oxitropium bromide is less known and less used than the previous ones but also acts as an anticholinergic bronchodilator for the treatment of asthma and COPD.

#### 3.1.9. Benzatropine

Benzatropine is a selective M1 muscarinic acetylcholine receptor antagonist with central nervous effects. Chemically, it is a combination of the tropine skeleton of atropine and the benzohydryl skeleton of diphenhydramine. It partially blocks cholinergic activity in the basal ganglia and increases the availability of dopamine by blocking its reuptake. This increases dopaminergic activity, therefore, it has found use in the treatment of early stages of Parkinson’s disease [43].

#### 3.1.10. Scopolamine and Its Use as an Antidepressant

Scopolamine may also be suitable for the application in central nervous system (CNS) diseases. It is known that scopolamine and other muscarinic receptor antagonists have an effect on the cognition processes, sensory functions (for example pain perception) and stress responses. As there is considerable evidence supporting the cholinergic-adrenergic hypothesis of mania and depression, the clinical effects of scopolamine as a central acting inhibitor of the muscarinic receptor has been tested. Several randomized double-blind studies have been performed and demonstrated contrasting outcomes. Some studies found scopolamine to have a rapid and prominent effect [44] while others found no benefit from scopolamine over placebo [45] for the treatment of these conditions. The contrasting findings indicate that more extensive studies are needed to verify the use of scopolamine for treatment of CNS diseases.

### 3.2. Cocaine Derived Drugs

Although cocaine has been used for a long time and by many people, little is known about it is use in treatment of neurobiology and pharmacology. The application of cocaine is legally restricted and consequently, the research is limited. It is known that cocaine exhibits different pharmaceutical modes of action like local anaesthetic properties, CNS stimulating actions and cardiovascular effects. However, these modes of action may alter according to the way of application—oral, nasal, by chewing, inhalation or by injection [46].

The central nervous effects such as euphoria, relief of fatigue and boredom as well as psychic stimulation are mainly explained by the resulting excess of dopamine after cocaine consumption. Cocaine inhibits the reuptake of dopamine, noradrenaline and serotonin, thus increasing their concentration in the synaptic cleft of the limbic system [8]. The intake of cocaine has an influence on the brain which is detectible in an electroencephalogram (EEG). However, the effects are inconsistent and may appear as increased or lowered signals in EEGs [47]. The local anaesthetic properties of cocaine by topical application are achieved by blocking the ion channels in neural membranes. Cocaine is absorbed by the mucosa after application and paralysis rapidly occurs in the peripheral ends of sensory nerves. It was widely applied in dentistry as a local anaesthetic but has been substituted by safer drugs. Nevertheless, it served as a lead substance for different local anaesthetics and painkillers. Procaine was the first major analogue of cocaine which was mainly used in dentistry. Nowadays, more potent local anaesthetics are available and so, its use has declined. A huge disadvantage of procaine is instability of the ester to hydrolysis. Tetracaine, a further development of procaine, is used for minor face surgeries and in ophthalmology. It is almost 10-times more potent than procaine, however, its toxicity increased proportionately to its potency [48]. Lidocaine is an amino amide analogue to the ester type of cocaine and was synthesized in 1943 by the Swedish chemists Nils Löfgren and Bengt Lundqvist [49]. Its advantages are the better stability towards hydrolysis in aqueous solution or esterase catalysis [48]. Beside its local anaesthetic properties, it is used as an Ib type antiarrhythmic medication due to its positive cardiovascular effects.

### 3.3. Calystegine Derived Drugs

Until now, no drug products derived from calystegines are available, although the inhibition of mammalian glucosides by these compounds may be a promising lead in the development of new active pharmaceutical ingredients.

## 4. TA Biosynthesis *In Planta*

### 4.1. Early Steps in TA Biosynthesis—A United Way

**Figure 3:**
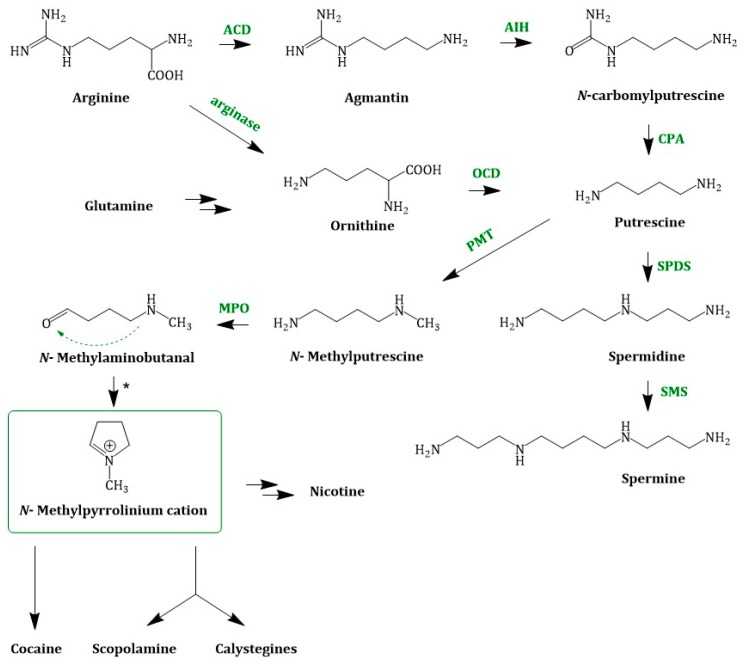
Joint steps of the early TA biosynthesis; ACD = arginine decarboxylase; AIH = agmatine deiminase; OCD = ornithine decarboxylase; CPA = *N*-carbamoylputrescine amidase; PMT = putrescine *N*-methyltransferase; SPDS = spermidine synthase; SMS = spermine synthase; MPO = *N*-methylputrescine oxidase; * = spontaneous cyclization.

The different classes of TAs: cocaine, scopolamine/hyoscyamine and the calystegines share a common precursor biosynthetic route (Figure 3) beginning with the amino acids l-ornithine (Orn) and l-arginine (Arg). In planta, Orn and Arg are formed from glutamate (Glu), an amino acid which is directly connected to the nitrogen assimilation. Ammonia (absorbed from the soil or synthesized from nitrate) is incorporated into Glu via the glutamine synthetase-glutamate synthase (GS-GOGAT) pathway. Glu is the precursor in several polyamine (PA) pathways. The regulation of PAs is very complex and not fully elucidated due to their pleiotropic functions [50] and PA concentration in plants can be used as indicators of various forms of abiotic stress.

In order to form putrescine (1,4-diaminobutane) from the amino acids Orn or Arg, Orn is decarboxylated by the ODC (ornithine decarboxylase; EC 4.1.1.17) and Arg undergoes a three-step reaction, including decarboxylation, hydrolysis of the imine functionality of guanidine and hydrolysis of urea which is catalysed by the enzymes ADC (arginine decarboxylase; EC 4.1.1.19), AIH (agmatine deiminase; EC 3.5.3.12) and CPA (*N*-carbamoylputrescine amidase; EC 3.5.1.53), respectively. The activities of ADC and ODC were suppressed in *Datura* plants by using the specific irreversible inhibitors dl-α-difluoromethylarginine and dl-α-difluoromethylornithine, respectively in order to probe the nature of these two routes to putrescine biosynthesis. These experiments indicated that the two routes do not act independently from each other and that the ADC exhibited a higher activity than the ODC [51]. Putrescine (tetramethylenediamine) is an intermediate in several metabolic pathways. It can be formed to spermidine by a spermidine synthase (SPDS; EC. 2.5.1.16) catalysed reaction using *S*-adenosyl methioninamine (decarboxylated S-adenosyl methionine) and putrescine as substrates.

Putrescine can also be methylated to *N*-methylputrescine by the enzyme PMT (putrescine *N*-methyltransferase; EC 2.1.1.53) [52] using SAM (*S*-adenosyl methionine). The next step in TA biosynthesis is the oxidative deamination of *N*-methylputrescine to 4-methylaminobutanal which is catalysed by a *N*-methylputrescine oxidase (MPO; EC 1.4.3.6) [53]. This diamine oxidase requires copper as a cofactor. *N*-methyl-Δ^1^-pyrrolinium, a central intermediate, is formed by spontaneous cyclization of *N*-methylputrescine. Chemically, this reaction is an intramolecular Schiff base formation. *N*-methyl-Δ*^1^*-pyrrolinium cation is a branchpoint in TA and nicotine biosynthesis [54]. In Figure 3. Joint steps of the early TA biosynthesis; ACD = arginine decarboxylase; AIH = agmatine deiminase; OCD = ornithine decarboxylase; CPA = *N*-carbamoylputrescine amidase; PMT = putrescine *N*-methyltransferase; SPDS = spermidine synthase; SMS = spermine synthase; MPO = *N*-methylputrescine oxidase; * = spontaneous cyclization, the joint biosynthesis is depicted. The condensation of nicotinic acid or more precisely its reactive derivate 2,5-Dihydropyrindine with *N*-methyl-Δ^1^-pyrrolinium cation yields nicotine.

### 4.2. Hyoscyamine and Scopolamine Biosynthesis

**Figure 4:**
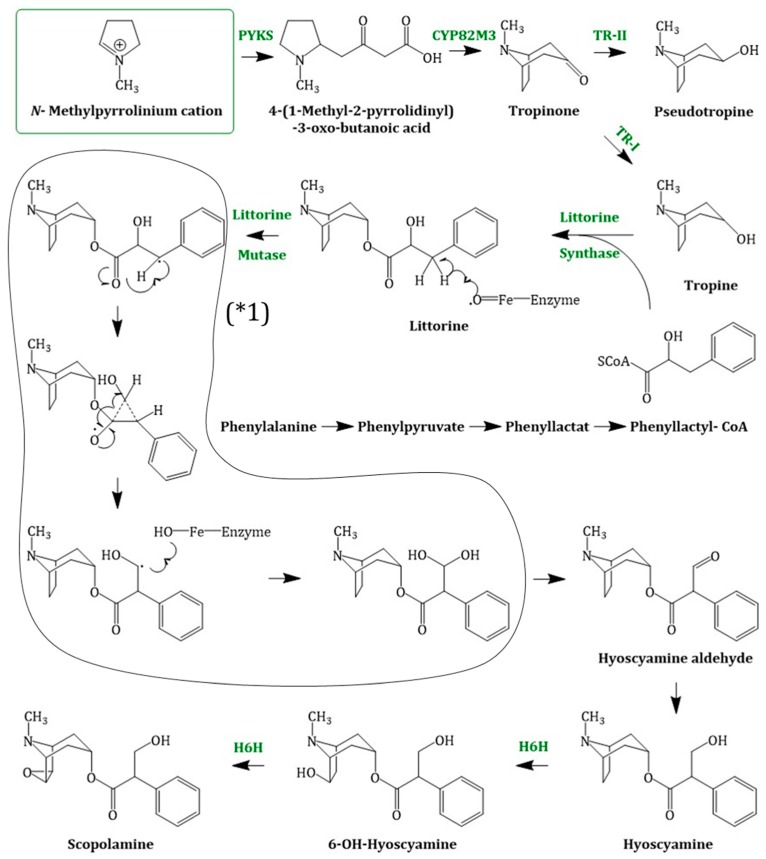
Scopolamine biosynthesis, starting with the *N*-methylpyrrolinium cation; PYKS = polyketide synthase; CYP82M3 = cytochrome P450 enzyme; TR-I/II = tropinone reductase I/II; littorine synthase (sequence not known); littorine mutase/monooxygenase (CYP80F1); (* 1) = proposed mechanism of littorine rearrangement; H6H = hyoscyamine 6β-hydroxylase.

Originating from *N*-methyl-Δ^1^-pyrrolinium, the next steps in the scopolamine biosynthesis (Figure 4) were not elucidated for a long time. Recently in 2018, Bedewitz et al. (2018) identified an atypical polyketide synthase from *A. belladonna* which catalyses the formation of the intermediate 4-(1-methyl-2-pyrrolidinyl)-3-oxobutanoic acid [55]. This intermediate is subsequently formed to tropinone by a malonyl-Coenzyme A mediated decarboxylative condensation catalysed by a cytochrome P450 enzyme, named AbCYP82M3. Tropinone serves as substrate for two stereospecific reductases: the tropinone reductase I (TR-I; EC 1.1.1.206) and the tropinone reductase II (TR-II; EC 1.1.1.236) [56]. TR-I catalyses its reduction to tropine (3α-tropanol), whereas TR-II catalyses tropinone reduction to pseudotropine (3β-tropanol), respectively. Pseudotropine is the precursor of calystegine biosynthesis while tropine is used to produce scopolamine.

Tropine is assumed to undergo condensation with activated (*R*)-phenyllactate (phenyllactyl-CoA), which delivers the third ring intermediate to littorine. Phenyllactate is derived from phenylalanine, an intermediate of the shikimate pathway, which is transaminated to phenylpyruvate. Bedewitz et al. (2014) discovered the coding sequence of a distinct aromatic amino acid aminotransferase (ArAT) that is co-expressed with known tropane alkaloid biosynthesis genes [57]. Silencing of ArAT4 in *A. belladonna* disrupted scopolamine biosynthesis by reduction of phenyllactate levels. The next step, the reduction of ketone function, is catalysed by a recently discovered phenylpyruvic acid reductase (*AbPPAR*). This reductase exhibited cell-specific expression also and was detected in root pericycle as well as the endodermis [58].

Although no enzymatic activity had been described, it is likely that an enzyme related to the cocaine synthase may be involved [59,60] in the formation of littorine. Littorine is rearranged via the littorine mutase/monooxygenase (CYP80F1; EC 1.6.2.4) to hyoscyamine aldehyde, which is subsequently reduced to the corresponding alcohol hyoscyamine [61]. Hyoscyamine is converted via the enzyme H6H (hyoscyamine 6β-hydroxylase; EC 1.14.11.11). The H6H is a 2-oxoglutarate dependent dioxygenase [62] which catalyses two reactions: first, the hydroxylation of hyoscyamine to 6β-hydroxy hyoscyamine and second, the epoxidation of 6β-hydroxy hyoscyamine to scopolamine. The bifunctional dioxygenase exhibits a strong hydroxylase activity in comparison to the rate limiting epoxidase activity [63].

#### 4.2.1. Enzymes Involved in Scopolamine Formation and Their Regulation

##### Putrescine Methyltransferase

The amino acid sequence of putrescine methyltransferase (PMT) is evolutionary related to those of plant spermidine and spermine synthases (SPMS, EC 2.5.1.22; converts spermidine into spermine; cofactor: decarboxylated S-adenosyl methionine). These enzymes are grouped in the spermidine synthase family by the Prosite database and contain a polyamine biosynthesis (PABS) domain. The PABS domain consists of two subdomains: I) *N*-terminal subdomain composed of six β-strands and II) Rossmann-like *C*-terminal subdomain [64]. It is assumed that PMT evolved from SPDS [65,66]. Teuber et al. (2007) performed a kinetic study of heterologous PMTs from different plants and measured K_cat_ values between 0.16 and 0.39 s^−1^.

##### Tropinone Reductase I and Tropinone Reductase II

Tropinone reductase I and II (TR-I (EC 1.1.1.206) and TR-II (EC 1.1.1.236)) are small proteins belonging to the short chain dehydrogenase/reductase (SDR) family and catalyze NADPH + H^+^-dependent conversion of tropinone into tropine or pseudotropine, respectively. They share the characteristic motifs of the SDR family, such as TGXXXGXG, a motif involved in NADPH binding, a NNAG motif and the catalytic sequence motif SYK [67]. Kushwaha et al. (2013) expressed the cDNA of *tr-I* from *Withania coagulans* in *Escherichia coli* and purified the protein to investigate its functional and catalytic properties [68]. They investigated the pH optimum, the thermostability, substrate saturations kinetics and specificity, as well as the effect of salts. A K_cat_ value of 16.74 s^−1^ for tropinone was determined. Additional work was performed by Qiang et al. (2016) on TR-I from *Brugmansia arborea* and *D. stramonium* [69]. The K_cat_ of BaTR-I for tropinone was 2.93 s^−1^ at pH 6.4 and the K_cat_ of DsTR-I was determined to be 2.40 s^−1^ at pH 6.4.

##### Putative Littorine Synthase

In 2015, Schmidt et al. published that the final step in the cocaine biosynthesis in *Erythoxylum coca*, the esterification of methylecgonine (2-carbomethoxy-3b-tropine) with benzoic acid, is catalyzed by a member of the benzylalcohol *O*-acetyl-, anthocyanin-*O*-hydroxycinnamoyl-, anthranilate-*N*-hydroxycinnamoyl/benzoyl- and deacetylvindoline 4-*O*-acetyltransferase (BAHD) family. This cocaine synthase is a plant acyltransferase, capable of producing both cocaine and cinnamoylcocaine via the activated benzoyl- or cinnamoyl-CoA thioesters. This esterification seems to be similar to the esterification of tropine with phenyllactic acid from scopolamine biosynthesis and hence, it can be assumed that the littorine synthase may also belong to the BAHD family. Enzymes of the BAHD family utilize CoA thioesters and catalyse the formation of numerous plant metabolites All identified members so far are monomeric, cytosolic enzymes with a molecular mass ranging from 48 to 55 kDa. The enzymes of this family share two conserved domains: The first is the HXXXDG domain, which is located near the center portion of each enzyme and is responsible for the utilization of CoA thioesters. The second highly conserved region is a DFGWG motif that is localized near the carboxyl terminus. These two motifs were identified in almost every functional enzyme of the BAHD family [70].

##### Littorine Mutase/Monooxygenase//CYP80F1

After the esterification of tropine with phenyllactic acid, the (*R*)-littorine formed is rearranged to (*S*)-hyoscyamine. Although the substrates for this isomerization were already identified in 1995 [71], the enzyme involved and its mechanism remained unknown until recently. Due to the similarity of this step to rearrangement reactions of comparable substances, it was speculated that this reaction is a coenzyme-B12 mediated isomerization. As no traces of vitamin B12 have ever been found in plants, this idea has been rejected [72]. Moreover, it was discovered that SAM is involved in the rearrangement of littorine to hyoscyamine. In 2006, Li et al. demonstrated in vitro that CYP80F1 (EC 1.6.2.4) converts littorine mainly to hyoscyamine aldehyde. Moreover, they showed that the suppression of the CYP80F1 gene by virus-induced gene silencing and RNAi results in the accumulation of littorine and reduction of hyoscyamine levels in planta.

##### Hyoscyamine 6β-hydroxylase

Hyoscyamine 6β-hydroxylase (H6H) is assumed to be the determining factor in many plants that accumulate hyoscyamine instead of scopolamine. H6H (EC 1.14.11.11) is a monomeric α-ketoglutarate dependent dioxygenase and the final enzyme of the TA pathway. This enzyme catalyses a two-step reaction, the hydroxylation of l-hyoscyamine to 6-hydroxy hyoscyamine and the epoxidation of 6-hydroxy hyoscyamine to scopolamine, exhibiting low epoxidase activity compared to hydroxylase activity [73]. The enzyme has an average molecular mass of 41 kDa and exhibits maximum activity at pH 7.8. l-hyoscyamine, oxygen and α-ketoglutarate are required for the enzyme activity, with respective K_m_ values of 35 µM and 43 µM. Iron ions (Fe^2+^), catalase and ascorbate (as a reductant) increase reaction catalysis. H6H is inhibited by EDTA and completely by other divalent cations, including Ca^2+^, Cd^2+^, Co^2+^, Cu^2+^, Hg^2+^, Mn^2+^, Ni^2+^, Zn^2+^, as well as by Fe^3+^. Several alkaloids which are structurally related to l-hyoscyamine have also been shown to be hydroxylated at the C-6 position of the tropane moiety by H6H. This enzyme also epoxidizes 6,7-dehydrohyoscyamine to scopolamine (K_m_ 10 µM) [74].

#### 4.2.2. Localization and Organization of Scopolamine Biosynthesis in Plants

The spatial localization of TA biosynthesis and their organization is diverse and complex. In Solanaceae plants, TA biosynthesis takes place in the roots and the alkaloids are then transported to the aerial parts where they are stored. Not much information regarding the transport and the transport form is available but it is assumed that the TAs are transported through the xylem. Cell-specific compartmentalization of scopolamine biosynthesis was previously observed in the root tissue pericycle, where expression of the genes *pmt* in *A. belladonna* [75] and *h6h* in *Hyoscyamus niger* [76] were detected. The enzyme TR-I, however, resides in the endodermis and nearby cortical cells in *H. niger* [56,77]. In potatoes, the TR-II, which provides pseudotropine for calystegine biosynthesis, was detected in the cortex and phloem parenchyma of roots and stolons; in tuber spouts, the protein was detected in companion cells. TR-I, whose function in potatoes is not yet elucidated, was also detected in protein extracts of tuber tissue, however, in quantities too low to permit localization to single cells [78]. The enzyme PMT also catalyses the first step in nicotine biosynthesis (discussed above). In *Nicotiana sylvestris*, a nicotine producing plant, *pmt* is expressed in the endodermis, outer cortex and found in root xylem [79]. This compartmentalization in biosynthesis in planta may complicate future attempts at heterologous production in single-celled microbial systems (discussed below). It may be that eukaryotic host cells such as yeasts or microalgae may be suitable host organisms for their biosynthesis as these cells exhibit compartmentalization of organelles and have been used for effective metabolic engineering of complex metabolites [80].

### 4.3. Cocaine Biosynthesis

**Figure 5:**
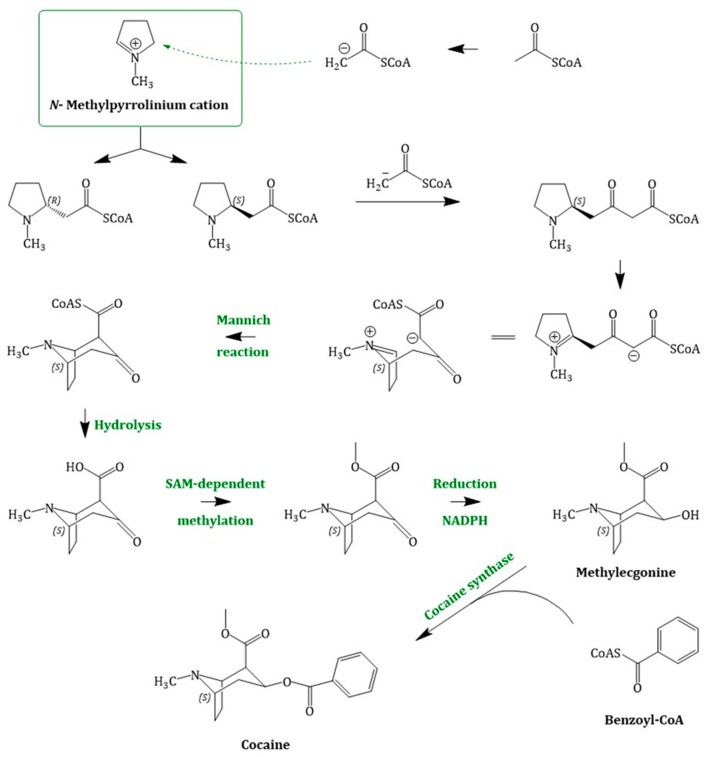
Cocaine biosynthesis, starting with the intermediate *N*-methyl-Δ^1^-pyrrolinium cation; only less information regarding the enzymatically involvement is proven. The iminium cation reacts with two acetyl-CoA moieties to an intermediate that cyclizes in an intramolecular Mannich reaction. After hydrolysis, methylation and reduction methylecgonine is formed. The cocaine synthase catalyses the last step in the pathway: the condensation of methylecgonine with benzoyl-CoA.

Cocaine biosynthesis (Figure 5), past its branch point with common intermediates shared with other TAs, is still under investigation and not fully elucidated. In literature, two different possibilities of the pathway towards cocaine biosynthesis have been reported. According to the classical hypothesis, the bridgehead carbon atom C-1 of methylecgonine is derived from an *N*-methyl-Δ^1^-pyrrolinium cation and that of C-2 originates from acetoacetate. However, feeding experiments with labelled *N*-methyl-Δ^1^-pyrrolinium cation were inconclusive in planta and could not confirm this theory. It was, therefore, suggested that the observed regiochemistry of incorporation of the labelled *N*-methyl-Δ^1^-pyrrolinium cation into cocaine was compatible with the stepwise introduction of C2 units into the ecgonine skeleton, derived from acetate [81]. Consequently, this hypothesis proposes a new intermediate in cocaine biosynthesis, *N*-methyl-2-pyrrolidineacetic acid. Although this compound was detected in several plants, all attempts at incorporation of it into the ester or thioester forms have been so far unsuccessful [82]. Chemically, nucleophilic addition of the first acetyl-CoA moiety reaction is assumed to be a Mannich-like reaction using the enolate anion; the side-chain extension occurs via Claisen condensation [48]. The (*S*)-enantiomer cyclizes and forms the bicyclic structure of the cocaine tropane ring skeleton by an intramolecular Mannich reaction [83]. Hydrolysis of the CoA-ester, followed by SAM-dependent methylation and reduction yield methylecgonine (2-carbomethoxy-3β-tropine). Methylecgonine in its turn condenses with benzoyl-CoA, which is derived from l-phenylalanine [84], to cocaine. Schmidt et al. (2015) described an enzyme catalysing this reaction and termed it the cocaine synthase. This synthase belongs to the BAHD family, which catalyses the transfer of CoA-activated acyl thioesters to oxygen- or nitrogen-containing acceptor molecules [70].

Cocaine and scopolamine differ mainly in two structures: First, the retention of the C-1 carboxyl group of acetoacetate, which is subsequently methylated and second, the different stereochemistry of the C-3 hydroxy group.

### 4.4. Calystegine Biosynthesis

Comparable to the hyoscyamine/scopolamine and cocaine pathways, the detailed processes of calystegine biosynthesis are not known. Tropinone is assumed to be involved and which should be reduced to pseudotropine, a reaction catalysed by TR-II. No further information regarding the biosynthesis is available so far [85]. To date, no attempts at elucidation of the hydroxylation or the demethylation of these compounds has been reported. This may be due to the relatively recent discovery of calystegines in 1990 [2] and reduced interest in medical applications compared to scopolamine or cocaine. In contrast to cocaine and scopolamine, the calystegine skeleton (8-azabicyclo [3.2.1] octane) is not *N*-methylated, rather, it is polyhydroxylated. These compounds are classified into different groups, depending on the number of hydroxy groups: subgroup A consists three, subgroup B four and subgroup C have five [86].

## 5. Biotechnological Approaches of Scopolamine Production and Alternative Methods of Raw Material Supply

### 5.1. Scopolamine Production in Cell Suspension and Hairy Root Cultures

The quality of *Duboisia* spp. plant material and the quantity of scopolamine in agricultural production depends on different abiotic factors such as climate, sunlight, soil fertilization and biotic factors [87,88]. In times of climate change, abiotic influences may become less predictable and more extreme. This in turn influences the biomass and results in variability alkaloid content and production potentials [89]. To establish a more independent production system, different plant cell cultures have been developed—especially callus cultures, cell suspension cultures and hairy root cultures. The advantage of these cell cultures is the possibility to control TA biosynthesis via process design in order to achieve increased or altered tropane alkaloid yields. However, to date, the produced amounts of TAs by tissue culture are not competitive to the production of scopolamine by agricultural farming of *Duboisia* hybrids. This difference in production arises due to complicated scale-up of tissue culture production and associated costs. Additionally, the cell-specific compartmentalization of TA biosynthesis as discussed in previous sections likely reduces tissue-culture specific production as callus and cell suspension cultures are totipotent, undifferentiated cells [90].

Hairy roots are disease manifestations developed by plants that are wounded and infected by *Agrobacterium rhizogenes* [91]. In contrast to undifferentiated cell cultures, hairy root cultures can usually synthesize the same metabolites as unmodified roots and may also produce desired secondary metabolites [92]. In nightshades, TA biosynthesis is localized in the root and this plant organ has been exploited for TA production. Early experiments were performed in the late 1980s and reports are continuing to be published on this process. The hairy root system itself is stable for several years with steady growth and alkaloid production rates [93], however, scale-up of this system remains technically challenging. Table 1 presents a brief overview of TA concentrations in engineered and untreated hairy root cultures from different plants:

A further development of the hairy roots cultures is the exploitation of TAs by “milking the plant.” After stimulation of aeroponically cultivated plants, roots were “extracted” by putting the roots into physiological extraction medium without harming or destroying them and desired secondary metabolites were isolated. After “milking,” plants are returned to their cultivation apparatus to regenerate and produce more secondary metabolites which can be subsequently extracted in further cycles. This promising approach still needs to be optimized to be economically competitive [105].

Transgenic plants have also been generated and cultivated for TA production. Recently, Xia et al. (2016) overexpressed *pmt* from *Nicotiana tabacum* (NtPMT) and *h6h* from *H. niger* (HnH6H) in *A. belladonna* and reached high scopolamine levels (2.94–5.13 mg g^−1^ DW) in field conditions [106]. Almost thirty years previously, Wang et al. (1985) also overexpressed *pmt* and *h6h* in *A. belladonna*, although a scopolamine concentration of only 1.2 mg g^−1^ DW was achieved [107].

To date, cell suspension cultures, callus cultures or hairy root cultures have been demonstrated to be competitive for TA production in comparison to agricultural means. In comparison to these alternative production options, the conventional field cropping of *Duboisia* species in Australia provides up to 15 tons fresh leaves per hectare, with three harvests annually. The total TA concentration of these plants is about 2–4% (equivalent to 20–40 mg g^−1^ DW) with ca. 60% scopolamine [1]. Obtaining these yields in terms of concentration and total amount is not yet competitive with biotechnological approaches.

### 5.2. Microbial Production of Scopolamine and Enzyme Engineering Approaches

Plants and plant cell suspension cultures are often slow growing and difficult to handle. In comparison, microbial cultures such as bacteria, for example, *E. coli* or yeast, for example, *Saccharomyces cerevisiae*, are straightforward to cultivate and are well characterized model organisms with fully developed molecular toolkits. Cultivation of these organisms can be readily scaled in existing fermentation infrastructure, which makes their cultivation more economically favourable than plant tissue culture. Therefore, heterologous production of TAs such as scopolamine in these hosts may represent an attractive alternative given transfer of the molecular pathways is possible.

Most research in this area has been performed on understanding and optimizing H6H by metabolic engineering. Cardillo et al. (2017) expressed recombinant *Brugmansia candida h6h* in *S. cerevisiae* and performed bioassays using isolated enzymes [108]. Untagged H6H was able to produce 83.3% 6β-hydroxy hyoscyamine and 7.6% scopolamine from hyoscyamine after 15 h of incubation. Additionally, specific hydroxylase and epoxidase activity: 2.60 ± 0.19 nKat mg^−1^ and 0.24 ± 0.02 nKat mg^−1^ for these two compounds were observed, respectively. The H6H from *Anisodus acutangulus* was cloned and expressed in *E. coli* fused with either a His- or GST-tag at the *N*-terminus [109]. A bifunctional assay revealed that both recombinant enzymes converted up to 80% of fed hyoscyamine to scopolamine, however, reaction kinetics were not analysed. Li et al., (2012) expressed *h6h* from *A. belladonna* (AbH6H) in *E. coli* and determined that the K_m_ value for hyoscyamine under optimal conditions was 52.1 ± 11.5 µM [110]. Compared with former experiments it revealed that the K_m_ of *Ab*H6H is higher than that of *Hn*H6H from *H. niger* (35 µM; [74]) and from *A. tanguticus* AtH6H (15.1 ± 0.3 µM; [111]), which implies that *Ab*H6H has lower affinity for the substrate than *Hn*H6H. Furthermore, it has been shown that epoxidation is slower than hydroxylation by this enzyme [63]. Pramod et al. (2010) characterized the H6H from *D. metel* and obtained K_m_ values for hyoscyamine and 2-oxoglutarate to be 50 µM each. In 2018, Fischer et. al., published results of SUMO-tagged H6H from *Brugmansia sanguinea* to have a K_m_ value of ~60μM [112].

First promising results concerning protein engineering of H6H were published in 2015. Cao et al. (2015) used random mutagenesis and site-directed saturation mutagenesis to increase the hydroxylation activity of H6H from *A. acutangulus* [113]. They developed a double mutant, *Aa*H6HM1 (S14P/K97A), which has a 3.4-fold increased hydroxylation and 2.3 times higher epoxidase activity than the native enzyme, a conversion rate of 97% was achieved in vitro.

The main challenge of the heterologous TA production is that the native biosynthesis of most target compounds in planta are not fully elucidated to date. It is not known if the condensation of tropine with phenyllactic acid-CoA reacts spontaneously or is enzymatically catalysed. Therefore, it is currently not yet possible to engineer the complete biosynthesis heterologously. The goal of microbial production of scopolamine is still in its early stages and will require complete pathway elucidation before it can be seriously considered as an alternative to conventional farming practices. Future efforts will require intense bioinformatic analysis of genomes and transcriptomic data to aid in identification of the complete biochemical pathways towards TA biosynthesis. Once identified, the pathways can be engineered into heterologous hosts and optimized for the generation of these desired products. More details on the application of bioinformatics in this field are discussed in Section 6.

### 5.3. Additional Methods of Scopolamine Production

To increase the scopolamine level in planta, polyploid plants have been developed TA yields determined. An impressive example was published by Dehghan et al. (2017) [114]. The authors produced stable tetraploid hairy root lines of *H. muticus* that exhibited lower biomass production than diploids, however, higher scopolamine (13.87 mg L^−1^) and hyoscyamine levels (107.7 mg L^−1^), up to 200% more scopolamine than in diploid plants. However, the total yield of scopolamine from these plants was rather low due to the slow growth rates and results were only reported for growth conditions in optimized Murashige and Skoog (MS) or Gamborg’s B5 media which are not competitive to conventional field cultivation. And moreover, other *Hyoscyamus* species like *H. senecionis* exhibit higher scopolamine then hyoscyamine levels in the leaves [115] which may be favourable for breeding and optimization approaches. Nonetheless, these initial trials are promising and polyploid plants of other species such as *D. myoporoides* may be interesting alternatives. In order for this strategy to realize economic potential, a polyploid clone must be able to be cultivated under the same conditions as the current plants, produce the same (or higher) biomass and be genetically is stable over a period of at least 10 years.

In 2017, Naik et al. published the first report regarding TA producing endophytes, namely *Colletotrichum boninense*, *Phomopsis* sp., *Fusarium solani*, *Colletotrichum incarnatum*, *Colletotrichum* siamense and *Colletotrichum gloeosporioides*, that are found in *D. metel* and possess the enzymes PMT, TR-I and H6H [116]. It was reported that these fungi produce a remarkable amount of scopolamine (4.1 mg L^−1^) and hyoscyamine (3.9 g L^−1^). Perhaps independent cultivation of these fungal species may represent a natural alternative to heterologous hosts or agricultural cultivation. It may also be possible to identify the biosynthetic pathways of TAs in these hosts, which could either be optimized in the fungi themselves and enhanced or transferred into heterologous microbial hosts.

## 6. Big Data—The Use of “Omics” in Plant Science and Pathway Elucidation

The biosynthesis of different TAs have not yet been fully elucidated. The further biosynthetic steps starting from the shared precursor *N*-methyl-Δ^1^-pyrrolinium cation are still uncertain. The latter stages of scopolamine biosynthesis are quite well elucidated, however, the formation of littorine is still unknown. Considerable progress was achieved in the elucidation of the cocaine biosynthesis by identification of the cocaine synthase, however many other steps in its biosynthesis have not yet been described. Calystegine biosynthesis is poorly investigated and consequently, very little information is known about its biosynthesis. Remaining metabolic steps may be elucidated through the concerted efforts of genomic, transcriptomic, metabolomic and proteomic data analysis, together “omics” coupled to biochemical and heterologous enzyme characterization.

The field of computational research is increasing and so are the potential applications and the ability to support laboratory investigations. With the help of powerful computing power, it is possible to process large amounts of data and filter relevant information. The four major disciplines—genomics, transcriptomics, metabolomics and proteomics—investigate the genetics, transcripts (messenger RNA/mRNA), complete proteins and metabolites in a biological sample, respectively. The four fields are connected indispensably: during protein biosynthesis or gene expression, which is a multi-layered process, two major steps are performed. In the first step, the information in DNA, the genes, is transferred to mRNA by transcription. The mRNA is then translated into proteins or peptides. Parts of these proteins are involved in different biosynthetic pathways, which produce then again different metabolites. The comprehensive data from these four disciplines are collected in a variety of ways but they come together in the sense that an evaluation can only be done with the help of computer science.

The genome of an organism usually remains constant throughout its lifetime; hence it is difficult to receive new information regarding biotic and abiotic regulation. The transcriptome, however, changes directly with the environmental conditions of the organism and so does the proteome and metabolome. This opens the possibility to use controlled lines of the appropriate organism and evaluation of its behaviour with respect to different parameters. Changing different parameters can for example provide information about gene regulation, help to elucidate biosynthesis, identify candidate genes or even possibilities for genetic engineering to enhance the production of desired metabolites. In literature, several examples for successful pathway elucidation using new techniques are described. Comparative transcript analyses identify candidate genes for secondary metabolism. In 2014, De Luca et al. reviewed how candidate genes for the monoterpenoid indole alkaloids (MIA) biosynthesis were identified by comparative transcript analyses [117]. Their roles in secondary metabolism were demonstrated by virus induced gene silencing and corresponding changes in metabolic profiles. Subsequently, these gene candidates could be heterologously expressed, purified and their biochemical roles characterized. Another example is the identification of candidate genes involved in betalain (tyrosine-derived pigments) biosynthesis. In this study, the authors combined transcriptomic analysis and metabolic profiling in *Mirabilis jalapa* and other betalain-producing species [118]. As these research fields are increasing, much more data also regarding other plants and the valuable secondary metabolites is available. As mentioned, the TA biosynthesis is not elucidated completely and important steps like the reaction of hygrine to tropinone as well as the synthase of littorine formation are not illuminated up to now. The application of “omics” with regard to the candidate gene isolation and the understanding of the process in detail will be enlightening.

## 7. Conclusions

TAs are a large and diverse group of plant secondary metabolites that can be divided into three major groups: hyoscyamine and scopolamine, cocaine and calystegines. The cultivation of coca, the source plant of cocaine, is illegal and no relevant pharmacological activity has been found for calystegine, therefore, only the cultivation of scopolamine containing plants is currently of legitimate economic interest. The demand for scopolamine is currently growing due to its various therapeutic applications and increasing market demand. Unfortunately, climate change and consequent variable abiotic and biotic stressors are resulting in challenges for agricultural production of this compound at high quantities in adequate quality. Investigations have been conducted in alternative production systems such as engineered plants, climate-independent production systems such as plant cell cultures or heterologous microbial production. To date, none of these alternative approaches has been economically competitive to the conventional scopolamine production from agricultural cultivation of *D. myoporoides* plants. The main issue preventing these new approaches from being successful is insufficient elucidation of the biosynthetic pathways towards different classes of TA biosynthesis. Not all enzymes have been described yet, the corresponding genes are unknown, rate-limiting steps have not been identified and the interaction of metabolic flux is not known. Newly available “omics” techniques will assist in the elucidation of these pathways, a strategy which has already been applied with other chemical classes from a variety of plant species.

Transfer of TA biosynthesis to heterologous hosts may present other challenges. For example, for some TAs, the production site in planta is not the storage site and compartmentalized production systems such as yeast or algal cells may be required to duplicate biosynthetic environments in microbial hosts. In addition, TA producing plant cells are specialized and only certain cells express their biosynthetic pathways, limiting the applications of undifferentiated plant-cell culture for TA production. Promising findings have recently been published which detail the potential for endophyte fungi to produce TAs and may be an interesting new source of these compounds separate from their plant hosts. Indeed, the production of scopolamine, in native plants or endophytes, in plant cell cultures or heterologously in microbial hosts is an exciting and dynamic field in which many new insights can be expected in the upcoming years, especially due to the application of modern “omics” technologies for pathway elucidation.

## Figures and Tables

**Table 1:** Overview of TA concentrations in engineered and untreated hairy cultures from different plants. TAs content in leaves of regenerated plants; n.d. = not determined, DW = dry weight.

Plant	Overexpression of	Amount		Citation
		Hyoscyamine	Scopolamine	
***Atropa belladonna***	-	0.371 ± 0.013% DW	0.024 ± 0.010% DW	Kamada et al., 1986 [94]
	*H. niger h6h*	0.02% *)	0.45% *)	Hashimoto et al., 1993b [95]
	*-*	2.1 + 0.2 mg g^−1^ DW	n.d.	Falk and Doran, 1996 [96]
	**)	0.31 mg g^−1^ DW	0.27 mg g^−1^ DW	Vakili et al., 2012 [97]
***Hyoscyamus niger***	*-*	1.6 mg g^−1^ DW	5.3 mg g^−1^ DW	Jaremicz et al., 2014 [98]
	*pmt, h6h*	n.d.	411 mg L^−1^	Zhang et al., 2004 [99]
***Anisodus acutangulus***	*h6h*	0.789 ± 0.078 mg g^−1^ DW	0.070 ± 0.003 mg g^−1^ DW	Kai et al., 2012 [100]
	*tr-I*	2.479 ± 0.432 mg g^−1^ DW	0.023 ± 0.004 mg g^−1^ DW	
	*tr-I, h6h*	2.286 ± 0.46 mg g^−1^ DW	0.072 ± 0.018 mg g^−1^ DW	
Brugmansia candida **)	*-*	0.35 ± 0.07 mg g^−1^ DW	1.05 mg g^−1^ DW	Cardillo et al., 2010 [101]
***Hyoscyamus muticus***	*h6h*	287.7 mg L^−1^	14.41 mg L^−1^	Jouhikainen et al., 1999 [102]
***Duboisia myoporoides***	*pmt*	no increase observed		Moyano et al., 2002 [103]
	*h6h*	n.d.	24.93 mg g^−1^ DW	Palazón et al., 2003 [104]

*) TAs content in leaves of regenerated plants **) chromium treatment ***) *B. candida* hairy roots grown in a special bioreactor.
